# Protocol for developing a model to promote the mental health of nurses caring for mental healthcare users at mental health institutions in Limpopo province, South Africa

**DOI:** 10.1371/journal.pone.0326360

**Published:** 2025-06-23

**Authors:** Annikie V. Hobyane, Hilda N. Shilubane, Tinyiko N. Rikhotso

**Affiliations:** Department of Advanced Nursing Science, University of Venda, Thohoyandou, South Africa; All India Institute of Medical Sciences - Patna, INDIA

## Abstract

The nurses as front liners caring for mental health care users (MHCUs) at mental health institutions are susceptible to adverse physical and psychological effects as a result of hostile behaviors of mentally ill patients. Most nurses suffer from stress that is caused by management’s lack of support, working overtime, lack of recognition by supervisors, and lack of skills and knowledge on how to handle aggressive mental health care users. The present study aims to develop a model to promote the mental health of nursing staff providing care to individuals with mental disorders at mental health institutions. Phase 1, the empirical face, will employ a qualitative, exploratory, and descriptive design focusing on nurses’ experiences caring for MHCUs and their views on improving their mental health. Information will be gathered using unstructured individual interviews with nurses purposefully sampled at selected mental health institutions. The study will apply procedures to safeguard trustworthiness and moral principles during the study. Phase 2 will focus on developing a model, and in phase 3, the developed model will be validated. The findings of the study will determine recommendations.

## Introduction

Globally, Nurses caring for MHCUs in mental health institutions have carried a heavy burden when caring for individuals with mental disorders, and the challenge to control the hostile behavior of MHCUs has directly faced its consequences [1]. The nurses, as front liners caring for MHCUs at mental health institutions, are susceptible to adverse psychological and physical consequences due to hostile behaviors of mental health care users [2]. Nurses are the backbone of the health care service as they are required to give nursing care to individuals with mental disorders 24 hours a day, regardless of users’ hostile behavior.

Studies show that most nurses suffer from stress due to management’s lack of support, working overtime without remuneration, as well as a lack of skills and knowledge on how to handle aggressive mental health care users [3; 4]. Similarly, research has demonstrated that most nurses who care for MHCUs develop symptoms of post-traumatic stress disorder, which is characterized by anger, anxiety [5; 6], reduced morale, burnout, increased absenteeism, decreased quality of care and work satisfaction [1; 7], depressive symptoms, denial, insomnia, and fear [5]. Some specific features of the hostile behavior of MHCUs may precisely increase their likelihood of affecting the nurses’ mental health (MH), which in turn impacts individual performance [8]. Consequently, it is essential to comprehend the nurses’ psychological needs to provide them with the necessary skills for alleviating the undesirable outcomes on their mental health.

In Bangladesh, nurses encounter psychological distress, higher workload challenges, exclusion, a lack of incentives, a lack of coordination, and organizational challenges during the provision of care to patients at mental health institutions [9]. Whereas in Iran, nurses rendering care to MHCUs at mental health institutions also experienced similar challenges of psychological distress with ineffective management support leading to high staff turnover, negative attitude towards the profession, as well as nurses leaving employment prematurely [10]. The authors also indicated that the mental health of nurses is a growing concern, as it directly impacts their well-being, work achievement, and the high standard of patient care. Some studies indicated that nurses rendering mental health care to MHCUs experienced increased psychological stress, and they were reluctant to go to work and considered resignation due to the absence of adequate support [11,12].

The lack of nurses’ recognition by management has had a significant impact on the psychological and physiological well-being of nurses as front liners and also increased concerns about raised suicidal behavior [13]. The quality of nurses’ sleep and warning signs of poor mental health, such as nervousness, strain, and depression, resulted in a constructive difference in the provision of care to MHCUs with hostile behavior [14].

Prolonged exposure to stress, long working hours, and emotional exhaustion can lead to burnout and mental health issues among nurses. The study conducted by Lee et al. [15] indicated that an extreme level of burnout, as well as psychological problems, were found to be linked to increased absenteeism, decreased job satisfaction, and decreased patient satisfaction. Therefore, maintaining nurses’ mental health should need to be an urgency [16]. Recent Indonesian statistics indicate that nurses could be accountable for between 10 and 20 percent of all psychological diagnoses, which might be an underestimation compared to highly industrialized and similarly impacted countries like Italy [17]. Mental health issues in healthcare professionals can occur due to a multitude of stresses, including the potential for getting sick, the concern of spreading the disease among friends and close relatives, inadequate availability of shielding clothing, and moral discomfort. [8].

A South African (SA) study by Baker and Naidu [18] examined the challenges of individuals with mental disorders in the primary healthcare setting. In contrast, Hobyane and colleagues examined nurses’ experiences caring for individuals with mental disorders suffering from the human immunodeficiency virus [19]. In Western Cape Province, professional nurses, enrolled nurses, and enrolled nurse auxiliaries caring for MHCUs at mental health institutions reported frustrations due to a lack of recognition by their supervisors [20].

In Limpopo province, several studies focused on MHCUs, for example, their perspectives concerning their family members’ participation in their care [21] and mental health care consumers’ experiences of mental health care [22]. Although a study conducted by Sono-Setati et al. [23] emphasized the significance of promoting sound minds and well-being among nurses to ensure not only their health but also the quality of healthcare delivery; there is still a gap regarding the enhancement of the sound minds of frontline carers of individuals with mental disorders in the provincial mental health institutions of SA. Thus, the current focus is to close this gap by developing a model to advance the sound mind of nurses who care for individuals with mental disorders at mental health institutions.

### Definition of terms

#### Mental health care users.

A mental health care user refers to “a person receiving care, treatment, rehabilitation services, or using health services at health establishments aimed at enhancing the mental health status of the user” [24]. In this study, MHCU means an individual admitted during the time of the study.

#### Mental health institution.

Facilities, residences, or buildings where MHCUs receive treatment, care, rehabilitation, and other health care services include health facilities like community care services, hospitals, health centers, rehabilitation centers, and mental health care institutions [24]. In this study, mental health institutions refer to specialized psychiatric hospitals in Limpopo province where MHCUs are admitted to receive care, treatment, and rehabilitation, that is, Hayani, Thabamoopo, and Evuxakeni.

#### Nurse.

A nurse is defined as “a person who is directed in the category of nursing under section 31(1) to practice the profession of nursing [25]. For the purpose of this study, a nurse means an enrolled nurse auxiliary (ENA), enrolled nurse (EN), and professional nurse (RN) working in specialized psychiatric hospitals during the time of the study.

#### Promote.

The term “promote” refers to assisting someone or something in moving forward.” It implies encouragement or fostering [26]. As part of this study, the term “promote” means the promotion of the mental health of nurses who care for MHCUs in specialized psychiatric hospitals during the time of the study.

## Materials and methods

### The study’s purpose and objectives

The study protocol intends to develop a model to improve the mental wellness of nurses who take care of individuals with mental disorders at mental health facilities in Limpopo province, South Africa. The research protocol’s objectives stem from the phases of the study.

### Phase one objectives

- To explore and describe nurses’ experiences when caring for individuals suffering from mental illness at mental health facilities in South Africa’s Limpopo Province.
- To examine the opinions of nurses providing care to individuals with mental health disorders in mental health institutions regarding the promotion of their psychological well-being.

### Phase two

The objective is to develop a model to improve the mental wellness of nurses caring for MHCUs at mental health institutions.

### Phase three

To validate the developed model.

### Theoretical framework

The theoretical framework defines the basic considerations of the study [27]. The PRECEDE-PROCEED model will guide the study [28]. The model provides an inclusive structure for identifying health needs and developing, implementing, and monitoring the promotion of health and other public health actions to meet the identified health needs. PRECEDE is a frame for creating a personalized and targeted health promotion program. PROCEED establishes a frame for implementing and evaluating public health interventions.

PRECEDE is an abbreviation for “Predicting, Reinforcing, and Enabling Constructs in Educational Diagnosis and Evaluation.” It includes evaluating the following immediate physical and social surroundings:

**Social Assessment:** Identify a population’s social problems, needs, and desired outcomes.

**Epidemiological Assessment**: Identify the health causes of the highlighted problem, which is the poor mental health of nurses, and establish priorities and objectives.

**Educational and Ecological Assessment:** Examine environmental and behavioral variables that contribute to the problem, support, and enable the specified behaviors and lifestyles.

**Health Program and Policy Development:** Determine and implement relevant interventions to promote the psychological well-being of nurses’ desired and expected outcomes.

PROCEED stands for “Policy, Regulatory, and Organizational Constructs in Educational and Environmental Development.” It comprises identifying intended results and implementing the model:

**Process Evaluation:** Determine whether the model reaches the intended audience, which is nurses, and achieves the anticipated outcomes.

**Evaluation:** Assess the changes in knowledge, capabilities, and attitudes and establish if there is a reduction in the incidence of poor psychological well-being in the nurses.

### Research methodology

The researcher uses the research methodology to perform the study and collect and analyze data pertinent to the research topic [29]. The study will use a qualitative method. A qualitative method is appropriate for this study because it will advance direct communication between the nurses and the researcher, resulting in a profound comprehension of the nurses’ experiences with care for individuals with mental disorders in mental health institutions.

### Design of study

The study design is an inclusive proposal for collecting information to respond to the identified problem [30]. It describes where, when, and how data will be collected and analyzed. This study will use a design that is exploratory and descriptive in nature to discover nurses’ experiences regarding the care of individuals with mental health disorders at mental health facilities and their views regarding improving their mental health [27]. It is suitable for this study as nurses can describe their lived experience while caring for MHCUs at mental health institutions and their views on how their mental health can be enhanced.

#### Exploratory design.

Through face-to-face individual interviews, the study will discover nurses’ experiences regarding caring for individuals with mental health disorders at mental health facilities/ mental health institutions in Mopani, Vhembe, and Capricorn Districts. In addition, the researcher will explore their views regarding the support needed to improve their mental health [31].

#### Descriptive design.

Nurses’ experiences regarding the care of individuals with mental health disorders at mental health facilities in one province of South Africa will be described. They will be provided the opportunity to express their views regarding the support to promote their mental well-being. Data will be collected through face-to-face, unstructured interviews with nursing personnel. Nurses will give thorough descriptions of the data concerning the nursing care they provide to individuals with mental health disorders. Furthermore, they will describe what needs to be done to enhance their mental well-being.

### Research setting

The research setting is a precise location for gathering information [32]. The setting for this study will be Limpopo psychiatric institutions where the nurses are caring for MHCUs with different mental disorders, such as “schizophrenia, bipolar mood disorder, depression, substance-induced psychosis,” psychogeriatric disorders, as well as intellectual disability. Different cultural groups surround the area, such as Xitsonga, Tshivenda, English, and Sepedi. This setting is found in the most rural areas. Most people in this area are unemployed and rely on social grants, pensions, and child support grants.

### Population of the study

The population describes the whole group of specific objects or individuals with similar characteristics. The study population will be nurses (professional, enrolled, and enrolled nursing Auxiliary) who render care to individuals with mental health disorders based on their job description and scope of practice. All these categories are allocated at mental health institutions.

In qualitative research, individuals with specialized knowledge or unique experiences with a particular occurrence are chosen to help the researcher better comprehend the topic [29]. The researcher in this study will select a portion of nurses as a sample.

## Sampling method and determination of sample size

Sampling will be done in three stages. A sampling of districts, a sampling of mental institutions, and a sampling of nurses.

### Sampling of districts

Districts will be purposefully selected for this study. The sampling of the districts is centered on the scholar’s judgement and knowledge of districts with specialized mental health institutions.

### Sampling of mental health institutions

Three mental health institutions will be purposefully sampled for this study. Their selection is based on the fact that they are the province’s only specialized mental health institutions.

### Sampling of nurses

Convenience sampling will be used to select the most conveniently accessible nurses as participants in a research project [29]. Convenient sampling will be used for nurses rendering care to MHCUs at mental health institutions. Accessible sampling will be all nurses allocated to the selected psychiatric institutions in Limpopo province.

#### Sample size.

In this study, we intend to conduct 36 interviews, 12 in each institution. Four registered nurses, four enrolled nurses, and four nursing auxiliaries will be interviewed in each institution. However, data saturation will determine the final sample size [33].

#### Inclusion criteria.

To qualify for participation in the study, participants must meet the admission requirements listed below.

- Be a registered professional nurse, enrolled nurse, or auxiliary nurse.
- They must be providing care to individuals with mental disorders admitted to the chosen mental health facilities providing care to individuals with mental health disorders.
- Should preferably be able to communicate in any of the following languages: English, Tshivenda, Sepedi, and Xitsonga.

#### Role of the researcher.

The researcher will utilize listening skills to promote successful conversations between the researcher and the nurses. Effective communication techniques include minimal spoken language, questioning, summarizing, explaining, interpreting, and paraphrasing. The researcher will write field notes whilst monitoring the nonverbal interview replies. Participants will be allowed to voice their thoughts about what they encountered while giving care to individuals with mental health disorders. The researcher will indicate the duration of the interview, which will last from 30 minutes to one hour. The interview will be conducted during lunchtime and recorded with a voice recorder at the three selected mental health institutions. No personal data will be associated with the recordings.

## Data collection

### Pre-test

The research questions will be tested for simplicity and clarity on nine nurses, three professional nurses, three enrolled nurses, and three auxiliary nurses with characteristics similar to the participants. Testing the nature of the questions will assist in modifying them if needed before the primary investigation. Pre-testing may also help estimate the duration of the interviews, which is anticipated to take 30–60 minutes, as indicated above, to align with the country’s 1-hour mandatory lunch break [34].

### Recruitment of participants

The researcher will visit three mental facilities in Limpopo Province’s selected areas and contact the nursing management to recruit subjects. When in the ward, ask the unit manager for permission to meet with the nurses. After nurses have been given complete information about the study, individuals who wish to participate will be requested to provide the researcher with their contact details. Researchers will contact the nurses to make arrangements to sign the consent forms before being interviewed.

The researcher will collect data using unstructured, in-depth interviews with nurses in private rooms during lunchtime to maintain privacy and avoid interruption of ward activities. The collection will last for three months. The following questions will guide the interviews:

*What is it* like to *care* for *MHCUs* at *mental health institutions?*

*What should be done to promote your psychological well-being?*

Probing questions will come after, depending on the responses of participants. The voice recorder will be utilized during the interview with the consent of the participants, and it can be terminated anytime on request by the participants (see Annexure A). Participants will be given number codes to protect their personal information.

### Data management

Data management refers to collecting, keeping, and storing data securely, cost-effectively, and efficiently [35]. For data safety, it will be backed up on OneDrive just in case the folder goes missing. The folder where the data will be kept will be protected with a password. Raw data will be presented to an independent coder to check whether themes are similar. Recorded data will be locked to ensure participants’ anonymity. Data will only be accessed by the research supervisors and the researcher and will be destroyed after five years of recording.

### Data analysis

Data analysis is the organization and combination of data research and interpretation. [36]. Individual verbatim responses will be transcribed. Data will be analyzed using Tesch’s eight steps [27]. The researcher and an independent coder will follow these steps to analyze data:

**Step 1**: Completely understand and read the entire text carefully. The researcher will prepare and organize the data that was collected. Notes will be transcribed verbatim.

**Step 2**: Select one transcript at a time and note the main point of each transcript.

**Step 3**: List all necessary elements and similar groups; List all points and group them. Divide the content into columns and group them into main content, unique content, and key content.

**Step 4:** Revisit the collected data with the list created in Step 3. Write the meanings in numbers and write the numbers next to the appropriate letters. Read again to see if new numbers appear.

**Step 5:** Determine the best descriptive terminology for the themes and organize them into categories. Look for ways to reduce the number of categories by grouping related themes. Create arrows within your categories to demonstrate connections between them.

**Step 6:** The scholar decides on the final abbreviation for each group and alphabetizes the codes.

**Step 7:** Gather the data for each category in one location and do an initial analysis.

**Step 8:** If needed, current data will be recoded. Following data analysis, a literature review is performed. Based on the findings from the examined data, recommendations will be created, including a description of how the model’s adoption will be assisted.

### Measure to ensure trustworthiness

Credibility, transferability, dependability, and confirmability are all critical components of trustworthiness in qualitative research.

### Credibility

Credibility is the accuracy with which outcomes convey the underlying meaning of data. Long-term participation in the data collection and triangulation process boosts credibility. To enhance the study’s credibility, the researcher will record in-depth interviews and take field notes [37].

### Transferability

Lincoln and Guba [38] employ transferability rather than generalization, implying that results in one context can be applied to similar people or settings. A detailed description of the participants’ demographics, research environment, and context will be supplied so that others can evaluate the transferability findings [38]. The researcher will employ in-depth interviews, long-term interaction, written notes, member verification, and triangulation.

### Dependability

Dependability implies that the study’s results must be reliable and precise for the study to be considered trustworthy. This necessitates an audit, which examines the techniques and processes followed by the scholar in the research and assesses if the findings are satisfactory, that is, reliable [29]. Dependability, thus, refers to the production evidence that, if replicated with identical or nearly identical individuals in the same setting, would yield the same results. This study will provide a detailed overview of the research methodology.

### Conformability

Polit and Beck [29] demand ‘conformability,’ meaning that a decision path or an audit should be done and accessible, where the person who reads can track the data sources. The results, recommendations, and conclusions should be reinforced by the data, and an internal link between the actual evidence and the investigator’s interpretation should be ensured. This is achieved by utilizing an audit trail.

#### Phase two: Model development.

According to Brink et al. [39], Conceptualization entails breaking down research topics into accepted meanings and establishing user consensus. The Phase 1 findings will be conceptualized in the PRECEDE-PROCEED model to develop a conceptual framework that will guide the development of the model.

The researcher will use the nominal group technique (NGT) to develop a model to promote the mental health of nurses caring for mental healthcare users at mental health institutions in South Africa. The NGT is a structured face-to-face stakeholder engagement to achieve consensus and action planning on a chosen topic [40], in this case, model development. The following steps of NGT will be followed:

1. Problem Identification and Preparation:
  - The session facilitator will introduce the question that is the subject of your brainstorming and ensure everyone understands the purpose and the nominal group technique process.
2. Silent idea generation:
  - All team members will be given time to think implicitly of solutions and ideas that come to mind regarding improving mental health and write as many as possible within a given time.
3. Round-Robin sharing:
  - Each panel member shares an idea while the session moderator records it on a flip chart. No explanation is permitted, and there are no questions about clarification.
4. Group discussion and clarification:
  - The panel members review the recorded ideas and are allowed to ask questions for clarity. Similar suggestions are combined, and duplicate entries are removed.
5. Voting and Prioritization:
  - Participants rank or vote on the ideas using a predetermined system (e.g., assigning numerical values). The solution with the highest overall ranking will be the final decision for inclusion in the model.

#### Phase three: Model validation.

After development, the model will be validated using the guidelines by Chinn and Kramer [41] described below.

Clarity: According to Chinn and Kramer [41], clarity is the ability of the theory to be clear and understood by its potential users.

Simplicity: When validating the model’s simplicity, the emphasis will be on the number of items in the model.

Generality: It refers to a model’s breadth of scope and purpose. The model will be examined to establish its applicability to nurses caring for MHCUs.

Accessibility: Determines to what extent the model’s aim can be met by seeking experts’ review.

The model’s importance relates to its clinical significance or practical worth.

The researcher will seek expert opinions on the developed model to promote the mental health of nurses who care for mental health care users. This method will help to ensure that the produced model is appropriate for the intended purpose. A panel of experts on the topic of study in Limpopo province (including external examiners, supervisor, co-supervisor, academics) will be permitted to evaluate the developed model based on its content and potential to improve nurses’ mental health.

## Ethical considerations

### Ethical approval

The University of Venda’s Human and Clinical Trials Research Ethics Committee approved the study protocol with the ethical clearance number FHS/24/PDC/05/0706. Permission to conduct the study will be obtained from the Provincial Department of Health in Limpopo province. All participants will be asked to provide their written consent. Information documents and consent forms will be provided. Participants who agree to participate in the study must read and sign this document. The authors are waiting for provincial permission to enter all three districts and their hospitals to gain access to research participants and begin data collection.

### Ethical principles

Dhai and McQuoid-Mason [42] identify four principles to examine when conducting research: autonomy, beneficence, justice, and non-maleficence.

### Principle of autonomy

This idea includes recognizing people’s right to make decisions based on their particular beliefs and values without the dominating influence of others. It also considers the individual’s right to autonomy and serves as the foundation for informed consent. The researcher is responsible for ensuring the research participants are not unfairly encouraged to participate [42]. They will be notified of all necessary information concerning the study, including the study’s goal, their protection of confidentiality, privacy, and the risk-benefit ratio, and that they will not be exposed to any harm, before giving consent. Written permission will be obtained for the interviews and audio recordings. The participants will decide the time for the interviews with the researcher. In this study, the researcher will inform the participants that a code or number will be provided to each of them or that they can devise their numbers or codes to ensure their identities are unknown.

#### Privacy.

The researcher will inform the participants that the interview will take place in a private room where there will be only the researcher and the participant to maintain privacy [30].

#### Confidentiality.

Confidentiality means that the information the researcher obtains from the research participants should not be shared with other people without their permission. Participants will be assured of the confidentiality of the interviews and that the recordings will be kept locked and accessible to the research team only [30].

#### Anonymity.

Anonymity means that the researcher should ensure that participant names will not appear in the study report. According to Gray et al. [32], the research process is ethical if it addresses issues such as anonymity. The researcher will inform the participants that a code or number will be provided to each of them or that they can devise their own numbers or codes to ensure their identities are unknown. In writing the report, the participants’ names will not be included.

### Principle of beneficence

The risk-benefit ratio will be discussed with the participants. There will be no direct benefit for the participant.

### Principle of non-maleficence

The principle of nonmaleficence states that one should not cause harm to others. As a result, the researcher will ensure that the participants are safeguarded from any injury or potential risk to their emotional well-being, private principles, and integrity during the process [42]. In this study, the researcher will ensure that the participants will not be vulnerable to risks more significant than those they are exposed to daily. When the participants experience discomfort, they will be referred to the hospital psychologist for counseling and debriefing.

### Principle of justice

In this study, the participants will always be treated equally by being given the same respect, for example, by being addressed appropriately. The subjects will be chosen for reasons directly connected to the issue being studied, not that they happen to be close by or easily influenced [43]. All participants will be allowed to seek clarity concerning the study questions. They will be informed about their right to discontinue participating in the study at any time if they are willing to do so, and will be notified of the study findings.

## Discussion

To the best of our understanding, no model has been developed to support the mental health of nurses who provide care to individuals with mental health disorders in the province. A strategy for promoting nurses’ mental health is significant because it can reduce anxiety, burnout, absenteeism, and suicidal behavior while increasing morale, care quality, and performance [44]. Individuals’ psychological well-being and resilience are crucial to preventing mental diseases and improving outcomes for those with them [45]. Although mental health concerns are frequent among nurses, the actual incidence of posttraumatic stress disorder (PTSD) among nurses remains unknown [46]. According to research, professional well-being is linked to job satisfaction, which includes finding meaning in work and having a high-quality work experience [47]. The current study attempts to develop a model to improve nurses’ psychological well-being in Limpopo province. It is considered that developing this model will enhance the emotional, psychological, and physical well-being of nurses and their capability to support the MHCUs.

## Conclusion

Our planned research will help us better understand the mental well-being of nurses caring for MHCUs at mental health institutions in Limpopo province. With this insight, the model will be developed to enhance the psychological well-being of nurses who care for MHCUs in mental health facilities. This, in turn, reduces the development of anxiety and suicidal ideation among nurses caring for MHCUs. Given the poor mental health of nurses caring for MHCUs in Limpopo’s mental health establishments, the model’s development will emerge as a critical instrument for improving the mental health of nurses caring for MHCUs in Limpopo’s mental health establishments.
